# Extensor Carpi Ulnaris Tendinopathy Following Conservative Treatment of a Distal Ulnar Metaphyseal Fracture: A Case Report

**DOI:** 10.7759/cureus.71027

**Published:** 2024-10-07

**Authors:** Takamasa Kudo, Akira Ikumi, Yuichi Yoshii, Yasukazu Totoki, Yukei Matsumoto

**Affiliations:** 1 Department of Orthopedic Surgery, Kikkoman General Hospital, Noda, JPN; 2 Department of Orthopedic Surgery, University of Tsukuba, Tsukuba, JPN; 3 Department of Orthopedic Surgery, Tokyo Medical University Ibaraki Medical Center, Ibaraki, JPN

**Keywords:** baseball, catcher, distal ulnar metaphyseal fracture, extensor carpi ulnaris, tendinopathy

## Abstract

Distal ulnar metaphyseal fractures are often treated conservatively with good clinical outcomes. Extensor carpi ulnaris (ECU) tendinopathy after conservative treatment for distal ulnar metaphyseal fractures has not been reported. We present a case of a 20-year-old university baseball player who developed ECU tendinopathy after conservative treatment for a distal ulnar metaphyseal fracture. A slight step-off at the fracture site and repeated movements as a catcher were considered to have caused the ECU tendinopathy. Clinicians should be aware of ECU tendinopathy after distal ulnar metaphyseal fractures with repeated ECU loading in sports or occupations.

## Introduction

Distal ulnar metaphyseal fractures are uncommon in isolation and are usually associated with distal radius fractures [1]. It has been reported that only 5-6% of distal radius fractures are accompanied by a distal ulnar metaphyseal fracture [1]. Isolated ulnar shaft fractures often result from direct trauma [2].

Usually, if pronosupination stress is repeatedly applied to the tendon for a prolonged period, extensor carpi ulnaris (ECU) tendinopathy may occur. Alternatively, ECU tendinopathy can also be induced by a recent injury [3]. In the setting of stenosing tenosynovitis or ECU tendinopathy, the initial treatment is primarily conservative with activity modification, anti-inflammatory medication, rest with a short course of immobilization, and trial steroid injection into the ECU tendon sheath [4]. Surgical decompression is reserved for chronic cases that have failed to improve after a minimum of two to three months of conservative treatment [4]. Depending on the cause of symptoms, surgical release or repair of the ECU subsheath may be performed [3].

However, ECU tendinopathy after conservative treatment of distal ulnar metaphyseal fractures has not yet been reported. Herein, we report the first case of ECU tendinopathy after conservative treatment of a distal ulnar metaphyseal fracture. A possible mechanism for this rare complication has also been discussed.

The patient was informed that data concerning the case would be submitted for publication, and he provided consent.

## Case presentation

**Figure 1:**
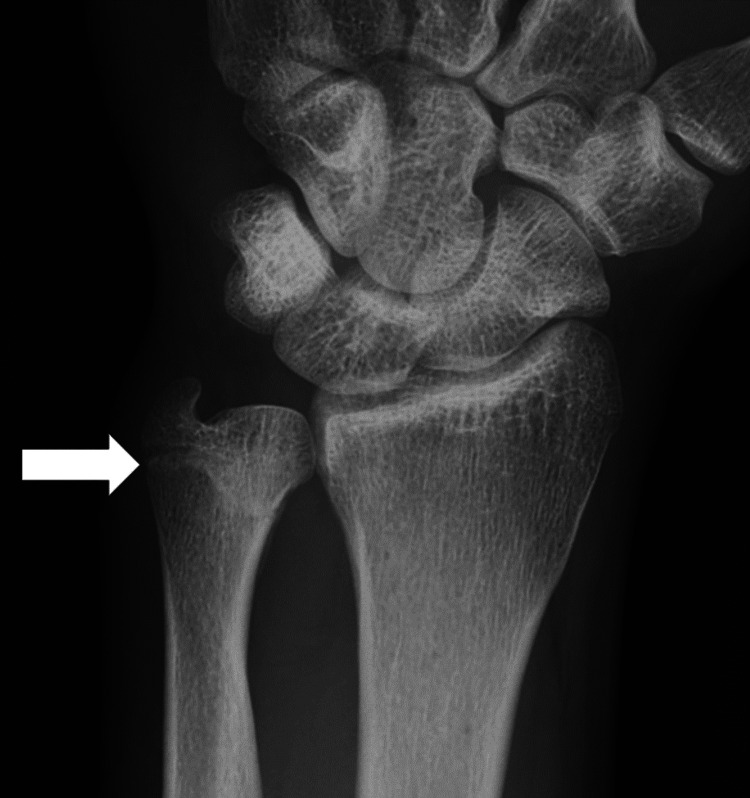
X-ray shows a fracture at the distal end of the ulna.

**Figure 2:**
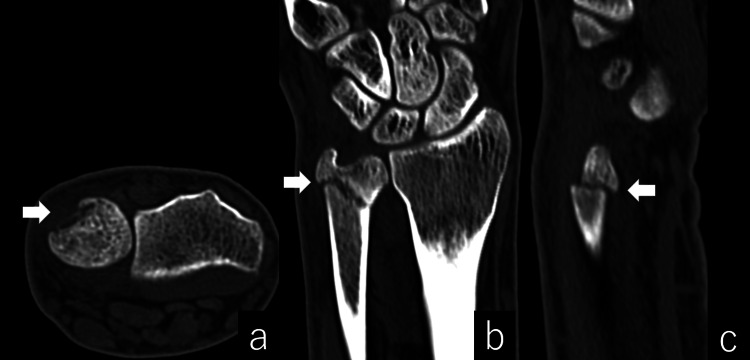
Computed tomography scan. Axial (a), coronal (b), and sagittal (c) views showed a 1-mm step-off on the dorsal side at the groove.

A 20-year-old university baseball player experienced a dead ball on the left wrist that fractured the distal end of the ulna (Figure 1). Conservative treatment with immobilization for four weeks was administered at another hospital. Computed tomography two weeks after injury showed a 1-mm step-off on the dorsal side at the groove (Figure 2). Two months later, bone fusion was achieved, and he attempted to return to playing baseball as a catcher. He had no difficulty in activities of daily living but was unable to play baseball due to pain on the ulnar side of the wrist and was referred to our institution. Four months after the injury, he could not catch a ball with the catcher's mitt because of pain in his wrist joints.

**Figure 3:**
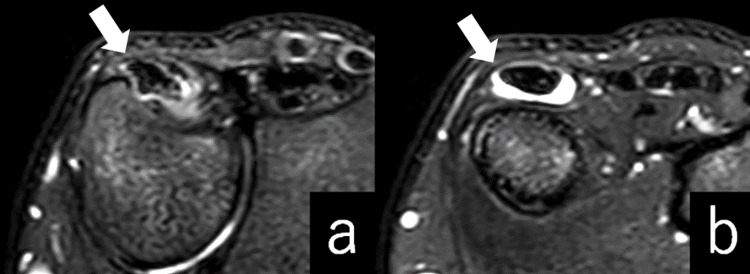
Preoperative T2-weighted fat-suppressed MRI showing the level of ECU groove. ECU tendon is deformed (a) and surrounded by reactive fluid or proliferated synovium (b). ECU: extensor carpi ulnaris.

Pronation, ulnar deviation, and supination were the cause of pain in the ulnar side of the wrist. Magnetic resonance imaging (MRI) revealed that the ECU tendon was deformed and surrounded by reactive fluid (Figure 3). Loss of contrast was not observed on ECU tendon sheath arthrography. Local anesthesia was administered to the ECU, distal radioulnar joint (DRUJ), and radiocarpal joint (RCJ) for the diagnosis of ulnar wrist pain to determine the degree of improvement in pain, and the patient had the greatest reduction in pain after the administration to the ECU. Based on these findings, we suspected ECU tendinopathy after the ulnar fracture and performed surgery.

**Figure 4:**
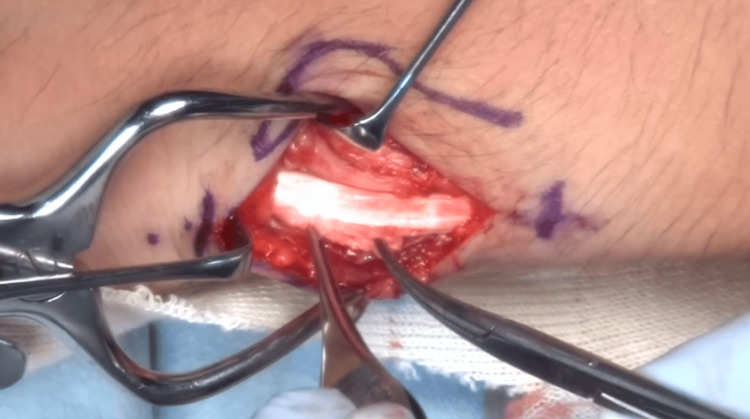
The tendon was partially damaged at the fracture site and covered with proliferated synovium.

**Figure 5:**
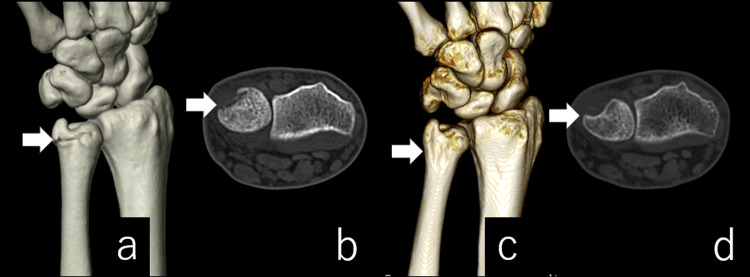
Computed tomography scan. (a) and (b) are CT images two weeks after the injury; (c) and (d) are postoperative images. The step-off at the fracture site was shaved and a groove was created.

Surgery was performed under general anesthesia. The ECU tendon was approached dorsolaterally. A straight incision was made over the ECU tendon that extended proximal to the ulnar shaft. The sixth extensor compartment was opened; however, there was no ECU subsheath. The ECU tendon was surrounded by reactive tenosynovitis, and a synovectomy was performed. The tendon was partially damaged at the fracture site (Figure 4). The step-off at the fracture site was shaved and a groove was created (Figure 5). After grooveplasty, smooth gliding of the ECU tendon was observed during wrist motion. As ECU tendon subluxation was not observed after grooveplasty, no subsheath reconstruction was performed and the incised extensor retinaculum was sutured in this case.

**Figure 6:**
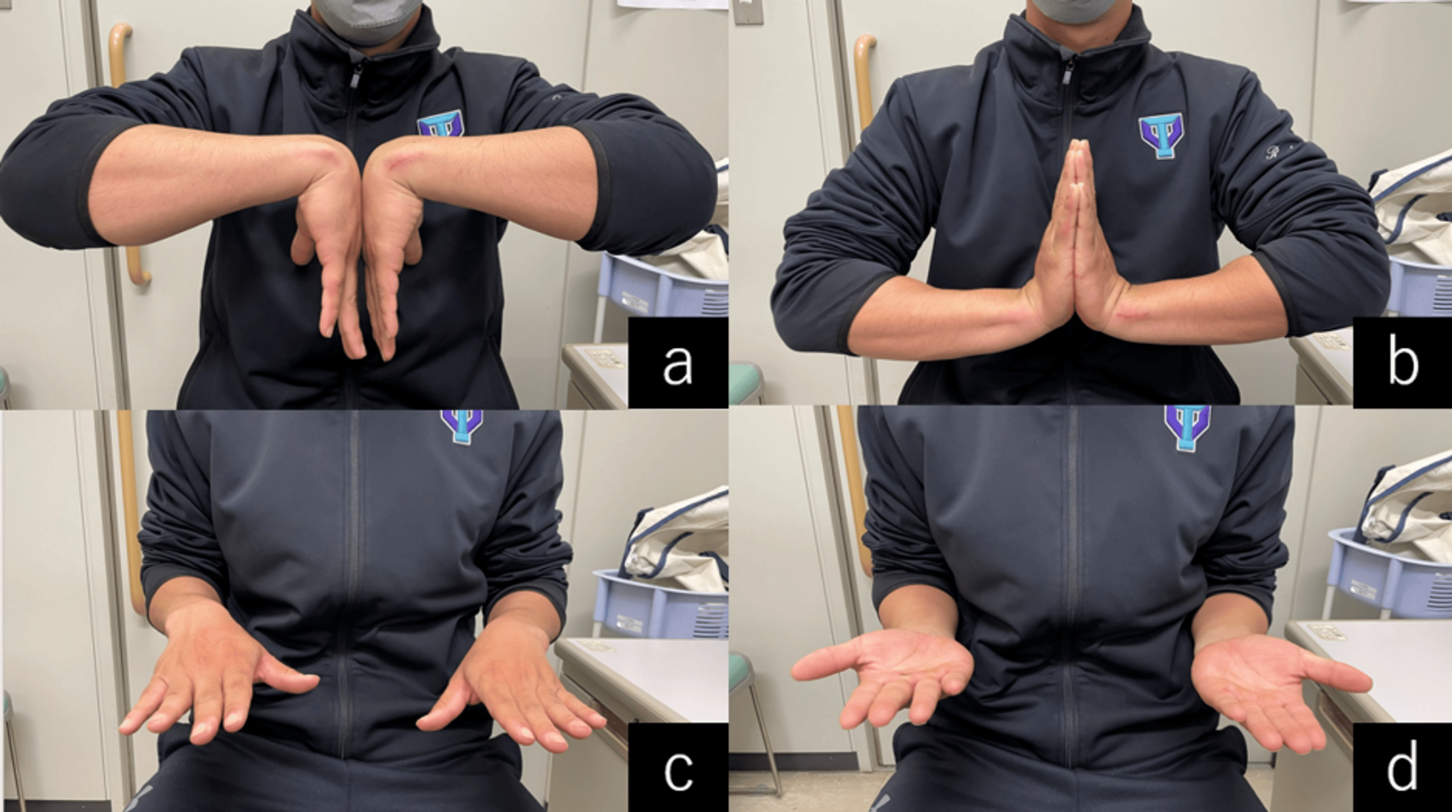
Six months after the operation, the active wrist joint flexion was 80° (a), extension was 90° (b), forearm pronation was 80° (c), and supination was 90° (d).

The postoperative course was uneventful with intensive hand therapy. The wrist was immobilized with a palmar splint for two weeks. Active range of motion was started at two weeks, and baseball was resumed in stages. He returned to playing baseball without any limitations two months after surgery. In the final follow-up six months after the operation, active wrist joint flexion was 80°, extension was 90°, forearm pronation was 80°, supination was 90°, and grip power was 55 kg (right: 60 kg) (Figure 6). The Quick Disabilities of the Arm, Shoulder, and Hand (DASH) score was 2.23 in disability/symptom and 6.25 in sports. The patient continued playing baseball without ulnar wrist pain recurrence.

## Discussion

Isolated distal ulnar shafts and metaphyseal fractures that demonstrate a displacement < 50% can be treated with casting. Fracture reduction and fixation are indicated for mid- and distal ulnar shaft fractures demonstrating > 50% displacement and over 10 angulations [5]. Therefore, the choice of conservative treatment at first admission in the present case was correct.

Chun & Palmer and Moran & Ruby described symptomatic rupture of the ECU tendon after trauma without fracture as the cause of post-traumatic ECU tendinopathy [6,7]. Hazel et al. considered after plating the distal ulna that a plate placed too far dorsally may cause irritation to the ECU tendon [8]. Also, malaligned fibrous nonunion of the ulnar styloid may cause impingement of the ECU tendon sheath [9]. Chun & Palmer described two unusual cases of symptomatic partial rupture of the ECU tendon associated with ulnar styloid nonunion [6]. However, ECU tendinopathy after distal ulnar metaphyseal fractures has not been reported.

The ECU tendon is held within its groove by the extensor retinaculum and its own separate subsheath in the distal 1.5-2 cm of the ulna [10]. In this case, the area covered by the subsheath was fractured. Surgical findings showed a partial rupture of the tendon just above the fracture site, but there was no ECU subsheath. The fracture was located in the area where the ECU was covered by the subsheath, and the formation of a step-off in its gliding floor was considered to have caused tendon wear and subsheath failure due to synovitis. In addition, the disappearance of symptoms after shaving the fracture gap suggests that fracture step-off was the cause of tendinopathy. However, it would have been unusual for step-off alone to cause ECU tendinopathy in this case.

The ECU muscle contributes variably to wrist flexion and extension, depending on the forearm position [11]. It is believed that tension on the subsheath is greatest during activities involving supination and holding the wrist in a flexed and/or ulnarly deviated position [10]. Sports-related ECU disorders are commonly observed in baseball, golf, hockey, and racquetball. The mechanism of injury is thought to involve forced wrist supination and flexion with ulnar deviation. Examples include an elite tennis player who torques the wrist in this position to maximize the topspin or the nondominant hand when performing a double-handed backhand. It can also occur during a check swing or the transition of ball contact to the follow-through phase while swinging a bat or golf club [12]. A unique feature of this case is that the pain was caused by catching a baseball. During pronation, the ECU serves as a pure ulnar deviator of the wrist [13]. The ECU is the only muscle that induces ulnar inclination when the wrist is extended [3]. Catching movements are performed with pronation and ulnar flexion at the extended wrist, which adds stress to the ECU. The catcher also immobilized or mildly spun the wrist into the catch position to ensure that the umpire's judgment was correct. When the wrist is immobilized, the ECU and flexor carpi ulnaris (FCU) are isometrically strained. Repeated movements apply loads on the ECU. The present case was a catcher, and the fact that this movement was frequent is considered to be a factor in the development of tendonitis despite the small fracture step-off. Although ECU tendinopathy in catchers is not often discussed in the literature, it has been reported that wrist injuries require the most rest among catchers, which should be given ample attention [14].

## Conclusions

We encountered a rare case of a 20-year-old university baseball player who developed ECU tendinopathy after conservative treatment for a distal ulnar metaphyseal fracture. The treatment of distal ulnar metaphyseal fractures requires careful follow-up and appropriate therapeutic intervention by identifying sports and occupations with repeated ECU loading at high risk of ECU tendinopathy development.
